# The GDP-Bound State of Mitochondrial Mfn1 Induces Membrane Adhesion of Apposing Lipid Vesicles through a Cooperative Binding Mechanism

**DOI:** 10.3390/biom10071085

**Published:** 2020-07-21

**Authors:** Andrés Tolosa-Díaz, Víctor G. Almendro-Vedia, Paolo Natale, Iván López-Montero

**Affiliations:** 1 Dto. Química Física, Universidad Complutense de Madrid, Avenida Complutense s/n, 28040 Madrid, Spain; antolosa@ucm.es (A.T.-D.); vgavedia@fis.ucm.es (V.G.A.-V.); 2 Instituto de Investigación Biomédica Hospital Doce de Octubre (imas12), Avenida de Córdoba s/n, 28041 Madrid, Spain

**Keywords:** mitochondria, membrane fusion, GTPase, mitofusin 1, giant vesicles

## Abstract

Mitochondria are double-membrane organelles that continuously undergo fission and fusion. Outer mitochondrial membrane fusion is mediated by the membrane proteins mitofusin 1 (Mfn1) and mitofusin 2 (Mfn2), carrying a GTP hydrolyzing domain (GTPase) and two coiled-coil repeats. The detailed mechanism on how the GTP hydrolysis allows Mfns to approach adjacent membranes into proximity and promote their fusion is currently under debate. Using model membranes built up as giant unilamellar vesicles (GUVs), we show here that Mfn1 promotes membrane adhesion of apposing lipid vesicles. The adhesion forces were sustained by the GDP-bound state of Mfn1 after GTP hydrolysis. In contrast, the incubation with the GDP:AlF${}_{4}^{-}$, which mimics the GTP transition state, did not induce membrane adhesion. Due to the flexible nature of lipid membranes, the adhesion strength depended on the surface concentration of Mfn1 through a cooperative binding mechanism. We discuss a possible scenario for the outer mitochondrial membrane fusion based on the modulated action of Mfn1.

## 1. Introduction

The fusion and fission of biomembranes are important events within the eukaryotic cell. The continuous remodelling of the organelle morphology such as the endoplasmic reticulum (ER) or mitochondria are essential to maintain cellular homeostasis and viability [1,2]. Membrane remodelling needs to overcome various energy barriers, where the target membranes must first be brought into very close proximity and then be destabilised to merge their lipid bilayers. Membrane merging occurs via a hemi-fusion intermediate structure in which the outer lipid monolayers are mixed whereas the inner monolayers remain unmixed [3]. Biological systems overcome energy barriers with the help of specialised proteins to control the efficiency of membrane fusion and fission. In the Endoplasmic Reticulum (ER), the fusion of membranes and tubules is mediated by the atlastin protein (ATL), a large membrane-anchored dynamin-like protein (DLP) of the dynamin superfamily [4]. ATL is next to dynamin one of the best studied DLPs [5]. DLPs are membrane active GTP hydrolysing proteins (GTPases) with high structural similarity, but low sequence identity that operate in different organelles within the eukaryotic cell where they trigger vesicle formation, membrane fusion, or organelle division [4,6,7]. ATL is a bifunctional protein that catalyses the docking (or adhesion) of the ER membranes through a GTP-dependent dimerization of its GTPase domain originating from adjacent membranes (*trans*), whereas the membrane fusion event is mediated by its carboxy-terminal (C-terminal) amphipathic helix that destabilises the lipid bilayer [8].

By analogy, mitochondrial fusion is controlled by the membrane-anchored outer mitochondrial membrane (OMM) DLPs mitofusin 1 (Mfn1), mitofusin 2 (Mfn2) [2,9] and the inner mitochondrial membrane (IMM) optic atrophy 1 protein (OPA1) [10]. Mfn1 and Mfn2 share a high homology of their amino acid sequence with an identity of 63.46% [11,12,13]. As ATLs, Mfns are transmembrane proteins with a large amino-terminal (N-terminal) GTPase domain, two heptad repeat domains (HR1 and HR2) suggested to arrange into coiled-coil structures (Cc1 and Cc2) and a hydrophobic region separated by five amino acids that might arrange into one [14] or two transmembrane segments (TMSs) [12]. Though the detailed mechanism of the Mfn-mediated membrane fusion is currently under debate [15], genetics indicate that the presence of a functional GTPase domain is mandatory [16]. Moreover, the available *holo* and *apo* crystal structures of truncated Mfn1 [17] together with the crystal structures of the bacterial DLP (BDLP) suggest that the GTP hydrolysis produces a power-stroke that changes Mfn from an extended four-helix bundle arrangement to a compact fold-knife like closed conformation [18,19]. Analogous to ATL, Mfn is hypothesised to approach adjacent *trans* Mfn-containing membranes into close proximity through this power-stroke and in this way to promote the membrane tethering and fusion [17]. The TMSs are absent in solved Mfn crystal structures, therefore their role during mitochondrial fusion remains speculative. In contrast, the TMSs of ATL were claimed to be critical for ER membrane fusion and shown to be organised as an intra-membrane hairpin rather than a bilayer-spanning [20]. Thus, for Mfn, the presence of one or two TMSs might be crucial to understand the correct protein topology and the molecular movement upon GTP hydrolysis. The power-stroke might be effected different if the C-terminal end of the protein faces either the mitochondrial intermembrane space or the cellular cytosol. To date, the accurate sequence of events remains hypothetical as only a few studies consider the use of the full length Mfn proteins [16,21].

Here we present the existence of a Mfn1-mediated membrane fusion intermediate. The full length human Mfn1 (*homo sapiens* Mfn1, *hs*Mfn1) protein was heterologously produced in *Escherichia coli* and isolated inner membrane vesicles were tested for membrane fusion activity. In vitro, we monitored membrane remodelling in the presence of GTP but we did not record any membrane fusion-like event. These data were extended by the use of palmitoyl-2-oleoyl-sn-glycero-3-phosphocholine (POPC) giant vesicles (GUVs) doped with 1,2-dioleoyl-sn-glycero-3-[(N-(5-amino-1-carboxypentyl)iminodiacetic acid)succinyl] (DOGS-NTA) lipids and *E. coli* membranes carrying histidine tagged *hs*Mfn1. Upon GDP or GTP incubation, we observed stable adhesion events of apposing vesicles. Surprisingly, in the presence of the GTP transition-state analogue GDP:AlF${}_{4}^{-}$, no adhesion events were observed. Therefore we suggest that the hydrolysis of GTP as well as bound GDP renders *hs*Mfn1 into an adhesion competent state. In addition, DOGS-NTA allowed to modulate the amount of histidine tagged *hs*Mfn1 present on the surface of POPC/DGS-NTA GUVs. We found that the increasing amounts of *hs*Mfn1 led to a stronger adhesion strength compatible with a cooperative protein–protein binding interaction. The increasing number of interacting molecules decreases the thermal fluctuation of the elastic membrane bilayer, which on its turn might promote and facilitate additional Mfn1–Mfn1 contacts from adjacent membranes [22].

## 2. Materials and Methods

### 2.1. Chemicals

Potassium chloride (KCl), glucose, sucrose, and 2-*Amino*-2-(hydroxy methyl)-1,3-propane diol (Tris), hydroxy-pyrene-1,3,6-trisulfonic acid (Pyranine), guanine-5-diphosphate (GDP) and guanine-5-triphosphate (GTP) were purchased from Sigma-Aldrich. n-Dodecyl-beta-D-maltoside (DDM) was purchased from Anatrace. Ultrapure water was produced from a Milli-Q unit (Millipore, conductivity below 18 MΩ cm).

### 2.2. Lipids

1,2-dioleoyl-sn-glycero 3-phosphoethanolamine (DOPE), 1-Palmitoyl-2-oleoyl-sn-glycero 3-phosphocholine (POPC), 1,2-di-(9Z-octa decenoyl)-sn-glycero-3-[(N-(5-amino-1-carboxy- pentyl) iminodiacetic acid) succinyl] (ammonium salt) (DOGS-NTA), *E. coli* total lipid extract (*E. coli* TLE) and the fluorescent probes 1,2-di oleoyl-sn-glycero-3- phosphoethanolamine- N- (lissamine rhodamine B sulfonyl) (Rh-PE) and 1-Oleoyl-2-[12-[(7-nitro-2-1,3-benzoxadiazol-4-yl)amino] dodecanoyl]-sn-Glycero-3-Phosphocholine (NBD-PC) were supplied by Avanti Polar Lipids and suspended in chloroform at 1 mg/mL and stored at −20 °C under nitrogen.

### 2.3. Cloning of Full-Length *Homo Sapiens* Mitofusin 1 (*hs*Mfn1)

The gene coding for the *homo sapiens* MFN1 gene (*hs*MFN1) was obtained by polymerase chain reaction (PCR) using template of total cDNA obtained from a human blood sample donated by Hospital 12 de Octubre, Madrid. The *hs*Mfn1 gene underwent PCR under standard conditions [23] using Taq polymerase (Roche), primers MFN1-FW( 5’-GCG GCA GCC ATA TGG CAG AAC CTG TTT CTC C) and MFN1-RV (5’ TGC TCG AGT GCG GCC GCA AGC TTG TCG ACT TAG GAT TCT TCA TTG CTT GAA GG) and a primer annealing temperature of 50 °C. The obtained PCR product was cloned into the pGEM-T easy (Promega) vector to construct plasmid pTL1 and was verified by DNA sequence analysis (UCM-Center for Research Support (CAI) Genómica y Proteómica, Universidad Complutense, Madrid). To construct His-Mfn1, the *hs*Mfn1 protein with an N-terminal histidine sequence extension under the control of a T7 promoter, the *hs*MFN1 gene was excised from pTL1 with restriction endonucleases NcoI and XhoI and transferred to the bacterial expression vector pET15 (Novagen) to construct pTL2.

### 2.4. Heterologous Production of hsMfn1 in Escherichia coli

The *E. coli* SF100 (DE3)-Codon plus-RP strain transformed with plasmid pTL2 (herein: SF100 (DE3)+/pTL2) was used to heterologous produce the histidine tagged full length *hs*Mfn1. This strain contains the Codon Plusp CP-RP plasmid (Stratagene) that codes for tRNAs *argU* and *proL* that recognises the arginine and the proline codons respectively of GC-rich mammalian genomes and necessary to produce human proteins in bacterial systems. The *E. coli* SF100 strain (*F-*, Δ*lacX74*, *galE*, *galK*, *thi*, *rpsL*, Δ*phoA*, Δ*ompA*), a derivative of the *E. coli* KS272 strain deficient for periplasmic OmpT protein (protease VII) [24], was further modified to *E. coli* SF100 (DE3) by the addition of the lambda phage DE3 cassette (λ DE3 Lysogenization Kit-Novagen, Merck Millipore) for protein production according to manufacturer’s instructions. DE3 allows the Isopropyl *β*-D-1-thiogalacto-pyranoside (IPTG) inducible production of T7 polymerase. For protein production, SF100 (DE3)+/pTL2 was inoculated overnight (ON) at 37 °C in LB growth medium [23] supplemented with 100 μg/mL ampicillin and 35 μg/mL chloramphenicol. The next day, the ON culture was diluted 1:100 in fresh LB supplemented with 0.2% glucose, 100 μg/mL ampicillin and 35 μg/mL chloramphenicol in a final volume of 1 L and incubated at 37 °C in an orbital shaker. At an optical density of 600 nm (OD_600_) of 0.6 culture was supplemented with 1 mM of IPTG and incubated for 1 h. Cells were collected by centrifugation (10 min at 10,000× *g*; Beckman A-10 Rotor; Beckman Coulter Allegra 25R Centrifuge), resuspended in 50 mM Tris-HCl pH 8.0 supplemented with 20% sucrose (w/v), flash frozen in liquid nitrogen and stored at −80 °C until further use.

### 2.5. Isolation of E. coli Inner Membrane Containing *hs*Mfn1

*E. coli* inner membrane vesicles (IMVs) and IMVs containing *hs*Mfn1 (Mfn1-IMVs), although with minor modifications, were essentially isolated as described in [25]. Thawed cells were broken by sonication (10 min; amplitude: 40%; 30 sec on/off cycles) with a Sonics Vibracell VC 750 tip sonicator (Sonics and Materials Inc. USA) in the presence of 1 mM PMSF (Sigma), 1 mM DTT (Sigma), 1 mM DNaseI (Sigma) and EDTA-free protease inhibitor cocktail (cOMPLETE; Roche). Low-spin centrifugation was performed on a Beckman–Coulter Allegra centrifuge (TA-10-250 rotor: 10 min at 10,000× *g*) and high-spin centrifugation was performed on a Beckman Optima-MAX-XP ultracentrifuge (MLA 80 rotor: 90 min at 327,578× *g* and TLA 100.3 rotor: 30 min at 264,499× *g*). Further characterisation of Mfn1-IMVs as Mfn1 protein stability and or protein orientation was checked by SDS PAGE [26] and Western blotting [27] using the Mfn1 specific monoclonal antibody Mfn1 D10 (sc-166644, Santa Cruz biotechnology Inc.) raised against amino acids 10–74 of *hs*Mfn1, mapping within an N-terminal cytoplasmic domain. The GTP hydrolysing activity of *hs*Mfn1 was checked by the malachite green phosphate assay [28] that detected inorganic phosphate liberated from GTP during GTP hydrolysis.

### 2.6. Preparation of Large Unilamellar Vesicles (LUVs) and Dynamic Light Scattering (DLS) Measurements

Liposomes were prepared according to the standard thin-film hydration method [29]. For the different formulations used here, the lipid chloroform solutions were mixed and dried using a vacuum concentrator (Eppendorf). The dried film was then hydrated with 500 μL of buffer solution (50 mM Tris HCl pH 8) and the final lipid concentration was 1 mg/mL. After vortexing for 10 min, the sample was extruded through a polycarbonate membrane (100 nm pore size; Avanti lipids). The diameters of the liposomes at different experimental conditions were measured by dynamic light scattering (DLS) in a 90 Plus Particle Analyzer (Brookhaven Instruments).

### 2.7. Total Lipid-Mixing Fluorescence Assay

To monitor the lipid mixing occurring between IMVs, carrying or not Mfn1, upon incubation with GTP, two sets of IMVs (fluorescent and non-fluorescent) were mixed together in a final volume of 100 μL. The ratio between fluorescent and non-fluorescent IMVs was 1:6 respectively leading to a total protein concentration of 1 mg/mL. Prior to mixing, the fluorescent set of vesicles was labelled with NBD-PC and RhoPE at a 1:5 molar ratio. At this ratio, the NBD group is quenched by the Rhodamine dye through Förster resonance energy transfer (FRET) [30]. The total concentration of dyes was lower than 1% mol with respect the lipid content. The fluorescence emission spectra (480–700 nm) were recorded for an excitation wavelength of 460 nm in a AMINCO Bowman Series 2 spectrofluorimeter before and after 60 min incubation with 10mM GTP. The FRET efficiency before and after GTP incubation was calculated through the ratio between the acceptor emission (at 593 nm) and the donor emission (at 523 nm). Control experiments were performed using LUVs made of *E. coli* TLE.

### 2.8. Electroformation of Giant Unilamellar Vesicles (GUVs).

Giant vesicles were prepared using the standard electroformation protocol [31]. The fabrication chamber was composed of two 1-mm spaced conductor indium tin oxide (ITO)-coated slides (7.5 × 2.5 cm^2^; 15–25 Ω/sq surface resistivity; Sigma). GUVs were prepared by transferring two 5 μL drops of 0.5 mg/mL of the lipid mixture to each ITO slide. After solvent evaporation, the fabrication chamber was sealed using Vitrex paste (Vitrex Medical, Herlev, Denmark). Then, the films were rehydrated in 200 mM sucrose solution and the electrodes of the chamber connected to an AC power supply (Agilent, 10 Hz, 1.1 V) for at least 3 h. When indicated, the lipid composition was supplemented with 0.5% of Rh-PE.

### 2.9. Confocal Fluorescence Microscopy

Confocal microscopy images of GUVs were collected at 22 °C with a Nikon Ti-E inverted microscope equipped with a Nikon C2 scanning confocal module, 488 and 561 nm continuous lasers, emission bandpass filters, and a Nikon Plan Apo 100x NA 1.45 oil immersion objective. The observation chamber was composed of two circular microscope cover slides (*ϕ* = 25 mm) spaced by a rubber ring (2.5 mm thickness). To avoid GUV adhesion or breakage, the bottom slide was incubated with 10 mg/mL BSA solution for 20 min prior to use. For observation, the chamber was extensively washed with 200 mM of sucrose solution. To bind *hs*Mfn1 via its N-terminal histidine tag to the NTA moiety of the DOGS-NTA lipid present on the surface of GUVs, we collected the detergent resistant membrane fraction of Mfn1-IMVs and extruded it through a 100 nm pore. Control experiments were performed with native *E. coli* IMVs that did not carry *hs*Mfn1. Then 90 μL of preformed GUVs were mixed with 10 μL of IMVs and incubated for at least 1 h at 22 °C before used. Subsequently the monoclonal Alexa Fluor 488 labelled Mfn1 Antibody (sc-166644 AF488) was supplemented to label the presence of Mfn1 on the GUV surface. When indicated, GUVs were imaged in the absence of fluorescent antibody, but in the presence of the fluorescent lipid probe Rh-PE, which was added to the lipid composition previously to electroformation. The confocal fluorescence images were captured with the Nikon NIS-Elements software and then further processed with ImageJ [32], where the fluorescence signal of the images were normalised to 50% of maximal intensities and the background fluorescence noise was reduced though the Image J despeckle function.

### 2.10. Quantification of the Adhesion Energy

The adhesion assay was performed by adding to different nucleotides (GTP, GDP or GDP:AlF${}_{4}^{-}$) and MgCl_2_ at a final concentration of 10 mM and 2.5 mM respectively. The magnesium concentration was not further increased to double its concentration with respect to the nucleotide concentration as it will cause the deformation and alteration of the GUV integrity and introduce experimental artefacts. Adhesion energies of Mfn1-GUVs in the presence of GTP or GDP were measured from the overall shape of adhered doublets as previously described [33]. Briefly, for each adhesion event, we acquired confocal microscopy images of the equatorial cross-sections and then further processed with the Nikon NIS-Elements software. The doublet contour of the GUVs was identified with MATLAB R2013b (The MathWorks Inc.). Adhesion energy densities *E_adh_* were calculated from the lateral tension and contact angles based on Young’s equation:

(1) $$E_{adh}=\sigma_{1}\left(1-cos\psi_{1}\right)+\sigma_{2}\left(1-cos\psi_{2}\right)$$

where $\sigma_{1}$ and $\sigma_{2}$ are the lateral tensions, and $\psi_{1}$ and $\psi_{2}$ are the contact angles. Lateral tension values were then directly calculated by inserting the reported bending rigidity of POPC-containing GUVs (κ = 10 k${}_{B}$T; [34]) in the correlation length $\xi_{\sigma }={\left(\kappa /\sigma \right)}^{1/2}$, obtained from fittings of the exponential function $y=\xi_{\sigma }^{\left(x-x_{0}\right)}/\xi_{\sigma }$ + y${}_{0}$ to the membrane contour, considered from the rim of the contact area up to the asymptote in the free membrane. This procedure was repeated for each adhesion event, thus obtaining an averaged adhesion energy density of all the adhesion events.

### 2.11. Cooperative Binding Adhesion

The adhesion strength can be analyzed in terms of the theoretical frame provided by the Dembo et al. [35]

(2) $$E_{adh}=k_{B}T\left[Mfn1_{A}\right]ln\left(1+\left[Mfn1_{B}\right]K_{2D}\right)$$

where $\left[Mfn1_{A}\right]$ and $\left[Mfn1_{B}\right]$ are the surface concentrations of Mfn1 in vesicle A and Mfn1 in vesicle B respectively, $k_{B}T$ is the thermal energy and $K_{2D}$ is the two-dimensional binding equilibrium constant defined by:

(3) $$K_{2D}=\left[Mfn1^{*}\right]/\left[Mfn1_{A}\right]\left[Mfn1_{B}\right]$$

where $\left[Mfn1^{*}\right]$, is the surface concentration of the Mfn1-Mfn1 complex. In the limit of $\left[Mfn1_{B}\right]K_{2D}\ll 1$ as expected for a weak adhesion regime, $E_{adh}$ is proportional to $K_{2D}$, i.e.,

(4) $$E_{adh}\approx k_{B}T\left[Mfn1_{A}\right]\left[Mfn1_{B}\right]K_{2D}$$

In contrast to rigid surfaces, the specific interactions through multiple Mfn1–Mfn1 membrane complexes, needs to suppress the repulsive membrane undulations of lipid bilayer counterparts. This implies that the two-dimensional binding equilibrium constant $K_{2D}$ is not constant but depend on the Mfn1 concentration [22]:

(5) $$K_{2D}\approx c\left(\kappa /\left(k_{B}T\right)\right)l^{2}\left[Mfn1_{A}\right]\left[Mfn1_{B}\right]{\left(K_{2D}^{{}^{\prime }}\right)}^{2}$$

where *c* is a numerical prefactor, κ a is the bending rigidity of vesicles, *l* is the binding range defined as the difference between the smallest and the largest local membrane separation at which Mfn1 proteins can bind and $K_{2D}^{{}^{\prime }}$ is a two-dimensional binding equilibrium constant for planar membranes at the Mfn1–Mfn1 optimal separation. Inserting Equation (5) into Equation (4) and considering that 3 NTA lipids can bind one Mfn1 molecule [36], leads to:

(6) $$E_{adh}\approx \left(c/81\right)\kappa l^{2}{\left(K_{2D}^{{}^{\prime }}\right)}^{2}{\left[NTA\right]}^{4}$$

### 2.12. Statistical Analysis

When indicated, Student’s *t*-test was performed to measure the significance of statistical difference between the different groups. p≤0.05,(∗) and p≤0.001,(∗∗∗) were considered statistically significant.

## 3. Results

### 3.1. The Heterologous Production of Human Mfn1 Protein in Escherichia coli

**Figure 1:**
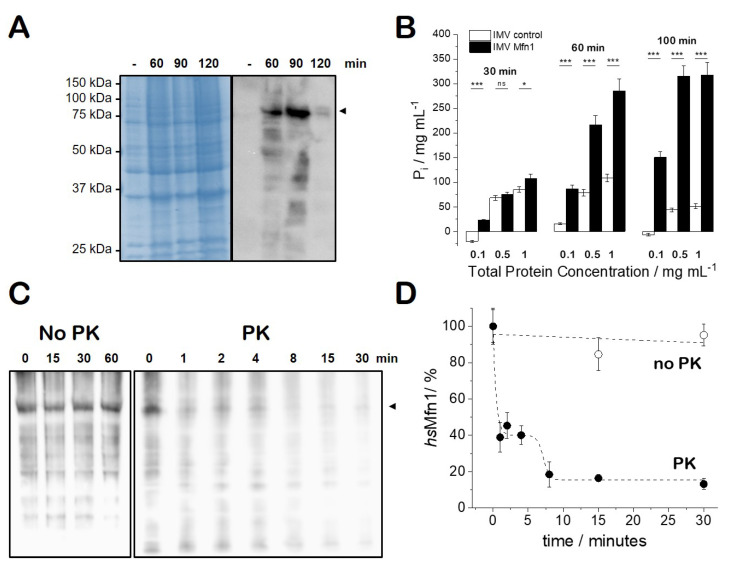
**Characterisation of histidine-tagged full length human Mfn1 protein produced in*****E. coli*****SF100(DE3) strain.** (**A**) 12% SDS-PAGE (left) and Western blot (right) with *E. coli* SF100(DE3)+/pTL2 cells before (-) or after (60, 90 120 min) after the addition of 1 mM IPTG. The arrowhead indicates the position of *hs*Mfn1 (∼ 82 kDa); (**B**) GTPase activity of Mfn1-IMVs supplemented with 10 mM GTP and incubated as indicated at 37 °C before developing with malachite green GTPase hydrolysis assay of *E. coli* IMVs in the presence of 10 mM of GTP. IMVs with a total protein concentration of 0.1, 0.5 and 1 mg/mL in the absence (white bars; control) and presence of *hs*Mfn1 (black bars) were incubated for 30, 60 and 100 min at 37 °C. Student’s t-test was performed to measure the significance of statistical difference between the different groups. p≤0.05,(∗) and p≤0.001,(∗∗∗) were considered statistically significant. (**C**) Protease protection assay of Mfn1-IMVs at a total protein concentration of 1 mg/mL in the absence (No-PK) or presence of 0.1 mg/mL of PK. IMVs No-PK were incubated at 37 °C and IMVs PK were incubated at 4 °C to be able to follow the breakdown. (**D**) Time evolution of protein breakdown as quantified from the protein band intensity during PK treatment.

The gene of the *homo sapiens* mitofusin 1 (*hs*Mfn1) protein was cloned and heterologously produced in *Escherichia coli* (*E. coli*). Plasmid pTL2 that carry the MFN1 under the control of T7 promoter was expressed in OmpT protease deficient strain *E. coli* SF100 (DE3). Though the overproduction of *hs*Mfn1 was not appreciated on SDS-PAGE (Figure 1A, left), Western blotting using the specific Mfn1 antibody showed that *hs*Mfn1 was present at 60 min and 90 min upon the induction with IPTG, but is then degraded at later time points (Figure 1A, right). We tried to optimise and maximise the production of *hs*Mfn1 by testing other growth conditions during protein production as the change of growth temperature, IPTG concentration or induction time, but we were not successful to obtain higher amounts of protein with the used strain, nor in other strains (*E. coli* BL21(DE3) or *E. coli* C41 (DE3) [37]) known to be optimised for the production of soluble and membrane proteins strains respectively. For the further experimental setup, we isolated *E. coli* inner membrane vesicles (IMVs) containing *hs*Mfn1 (Mfn1-IMVs).

### 3.2. GTP-Hydrolysis of E. coli IMVs Carrying *hs*Mfn1

We tested the ability of Mfn1-IMVs and IMVs that did not carry Mfn1 for GTP hydrolysing (GTPase) activity. We used IMVs at a total protein concentration of 0.1, 0.5 and 1 mg/mL resuspended in assay buffer (50 mM Tris HCl pH 8; 1 mM MgCl_2_) and incubated for 30 min at 37 °C in the presence of either 2 mM or 10 mM of GTP. GTPase activity detected through malachite green assay [28] that detected the amount of inorganic phosphate released upon GTP hydrolysis. Compared to the control IMVs, we observed a stimulated GTP hydrolysis of Mfn1-IMVs when incubated with 10 mM GTP (Figure 1B). No significant GTP hydrolysis was observed when IMVs were incubated with 2 mM of GTP (data not shown). We observed a correlation of the the amount of P*_i_* produced with the amount of total protein tested (Mfn1-IMVs) and the increase in incubation time (30, 60 and 100 min). At 100 min the amount of GTP in the sample must have been depleted as no significant increase of the amount of P*_i_* was observed with respect to 60 min of incubation. These results suggest that the heterologously produced *hs*Mfn1 was proficient for GTP hydrolysing activity, but as we needed a high concentration (10 mM) of nucleotide to observe the GTP hydrolysis we must conclude that our *hs*Mfn1 protein either bound or hydrolyzed the nucleotide with a low affinity.

### 3.3. Characterisation of E. coli IMVs Carrying hsMfn1

To check if *hs*Mfn1 was routed to the cytoplasmic membrane of *E. coli*, we fractionated the broken histidine-tagged Mfn1 protein-producing *E. coli* SF100(DE3)+ cells and identified the presence of *hs*Mfn1 in the isolated cytoplasmic membrane or inner membrane vesicles (IMV). We checked our full length *hs*Mfn1 for orientation within or IMVs to see if this material was suitable for membrane fusion experiments. The current model states that for membrane fusion the GTPase domains of adjacent membranes must face each other and that upon direct interaction and GTP hydrolysis membrane fusion will take place [21]. We expected the orientation of the membrane and membrane proteins within the IMVs to be orientated in a scrambled orientation as we used sonication for cell disruption [38]. First we incubated Mfn1-IMVs up to 1 h in 50 mM Tris HCl pH 8, 50 mM NaCl in the absence of proteinase K (PK) to test the protein stability within an experimental time frame that should lead to membrane fusion. The presence of *hs*Mfn1 was checked by Western blotting. No degradation of *hs*Mfn1 was observed (Figure 1C, left). Then, we incubated Mfn1-IMVs at 4 °C in the presence of 0.1 mg/mL PK (Figure 1C, right). At 4 °C PK activity was moderated and under this condition we could detect a difference in protein profile and avoid the immediate collapse of the Mfn1-IMVs. Within the first four minutes we observed a 60% reduction of the Mfn1 signal, whereas the total protein profile of the background did not significantly change (Figure 1D). Longer incubation times deteriorated the total protein profile (80% degradation of *hs*Mfn1 after 8 min and 85–90% degradation of *hs*Mfn1 after 15 to 30 min) (Figure 1D). Eventually the PK collapsed the IMVs and digested all proteins present in the membrane. This experiment roughly indicated that *hs*Mfn1 was oriented approximately 50:50 with its large N-terminal soluble domain facing both sides of the membrane. Here we could not conclude if the N- and C-terminal domain of Mfn1 faced the same or opposite sites of the membrane. These results indicate that our material is suitable for in vitro fusion experiments as the *hs*Mfn1 protein that was oriented to the external face of the membrane will participate in the fusion process.

### 3.4. Lipid Mixing Assay of Mfn1-IMVs

**Figure 2:**
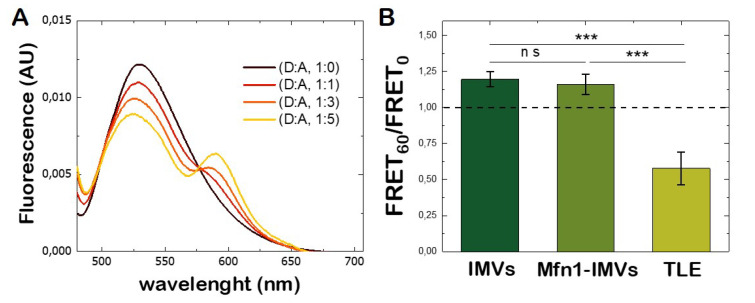
**Total lipid mixing assay of inner membrane vesicles (IMVs).** (**A**) Fluorescence emission spectra of IMVs labelled with NBD-PC (donor, D) and RhoPE (acceptor, A) at a different molar ratios. The excitation wavelength was 460 nm. The peaks at 523 and 593 nm correspond to the maximum fluorescence emission of the donor and acceptor respectively. An optimal Förster resonance energy transfer (FRET) from NBD to Rhodamine was observed at molar ratios of 1:5 between the donor and the acceptor. (**B**) FRET efficiency ratio of IMVs (left), Mfn1-IMVs (middle) and *E. coli* total lipid extract (TLE) large unilamellar vesicles (LUVs) (right), before and after 60 min incubation with 10 mM GTP. Student’s *t*-test was performed to measure the significance of statistical difference between the different groups. p≤0.05,(∗) and p≤0.001,(∗∗∗) were considered statistically significant.

The fusion between lipid membranes was accompanied with the mixing of the lipid content present on both bilayers, so that the lipids from one membrane were able to diffuse into the other membrane and vice versa. A FRET-based assay is typically used to monitor the lipid exchange [39]. The principle consists of labelling one population of membranes with lipid fluorescent probes acting as donor and acceptor at sufficient concentration to produce FRET. Figure 2A shows IMVs labelled with NBD-PC (donor) and RhoPE (acceptor) at different molar ratios. An efficient FRET signal from the NBD dye to the rhodamine dye was found at a molar ratio of 1:5 respectively. The fusion of labelled IMVs with non-labelled IMVs leads to a dilution of fluorophores present of the labelled membranes by increasing the distance among them and a decrease the FRET efficiency was monitored upon excitation of the donor probe. Because the presence of *hs*Mfn1 might have led to vesicle fusion upon GTP hydrolysis, we monitored the fusion between Mfn1-IMVs and compared the result with fusion of IMVs in the absence of Mfn1. Surprisingly, for both cases we observed an average increase of 20% of the FRET efficiency (Figure 2B). The absence of an expected FRET decrease for *hs*Mfn1-carrying membranes suggested the existence of membrane remodelling effects, such as aggregation, rather than membrane fusion. To support this interpretation, we also monitored, as a control experiment, the spontaneous membrane fusion of LUVs made of *E. coli* total lipid extract (*E. coli* TLE) in the presence of 10 mM GTP and the absence of proteins. In this case, the observed decreased FRET signal within the measured experimental time-scale was associated with the dilution of NBD-PC and RhoPE concentrations as the result of spontaneous membrane fusion (Figure 2B).

### 3.5. Specific Binding of hsMfn1 Protein to the GUVs

**Figure 3:**
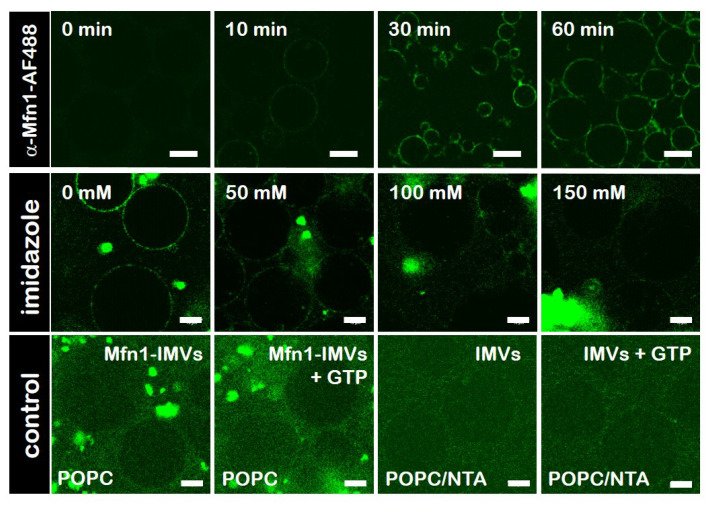
**Specific binding of*****hs*****Mfn1 protein to giant unilamellar vesicles (GUVs)**. Confocal fluorescence microscopy images showing the time series of the binding (**top row**) and release (**middle row**) of α-Mfn1-488 antibody to *hs*Mfn1 immobilised on the surface of POPC/DOGS-NTA GUVs. Prior to the incubation with POPC/DOGS-NTA GUVs, *hs*Mfn1 itself was incubated with α-Mfn1-488 (green signal). To release the bound *hs*Mfn1 from the POPC/DOGS-NTA GUVs, increasing amounts of imidazole were added to the sample. Control assays (**bottom row**), where POPC GUVs (in the absence of DOGS-NTA) were incubated with Mfn1-IMVs (**bottom row**, Mfn1-IMVs) or POPC/NTA GUVs were incubated with solubilised *E. coli* IMVs (**bottom row**, IMVs) showed no labelling of the GUV contour upon incubation with α-Mfn1-488. To visualise the presence of GUVs in the fluorescence images of the control experiments, the raw data were not corrected for signal-to-noise ratios. The addition of GTP to these samples did not promote adhesion of vesicles. Scale bars are 10 μm. Each control was repeated independently at least three times.

To visualise differences in membrane remodelling events mediated by *hs*Mfn1, we used giant unilamellar vesicles (GUVs) composed of POPC mixed with DOGS-NTA at a mol% ratio of 80 to 20, respectively. GUVs were decorated with the detergent resistant fraction of Mfn1-IMVs. To confirm the specific binding of *hs*Mfn1 to the DOGS-NTA moiety on the POPC/DOGS-NTA GUVs surface we used the monoclonal Alexa Fluor 488 conjugated Mfn1 Antibody (α-Mfn1-488). The incorporation of the histidine-tagged Mfn1 on the lipid surface of GUVs (*hs*Mfn1-GUVs) was monitored by confocal fluorescence microscopy as an increase in the fluorescent signal of the vesicle contour (Figure 3, top row). We observed a dotted fluorescence signal along the contour of vesicles, which kept their spherical shape with a variable size ranging from a few up to several tens of μm. The addition of imidazole up to a final concentration of 100 mM cleared the sharp fluorescent contour of the GUVs (Figure 3, middle row). Imidazole disrupts the interaction of the DOGS-NTA lipid and the histidine residues of the *hs*Mfn1 protein and the dislodged protein (and its fluorescent label) returned into the buffer solution. An additional proof for the specific interaction between histidine-tagged Mfn1 and NTA lipids was obtained by incubating POPC GUVS (without DOGS-NTA) with *hs*Mfn1 and its fluorescent antibody, as well as POPC/DOGS-NTA GUVs with solubilised native *E. coli* IMVs that did not contained *hs*Mfn1 and the fluorescent Mfn1-antibody. Under these conditions we did not obseve a discrete fluorescent contour of GUVs but rather a weak, fuzzy and diluted background signal of the buffer (Figure 3 bottom row).

### 3.6. The GDP-Bound State of Mfn1 Promotes the Adhesion of Lipid Vesicles

**Figure 4:**
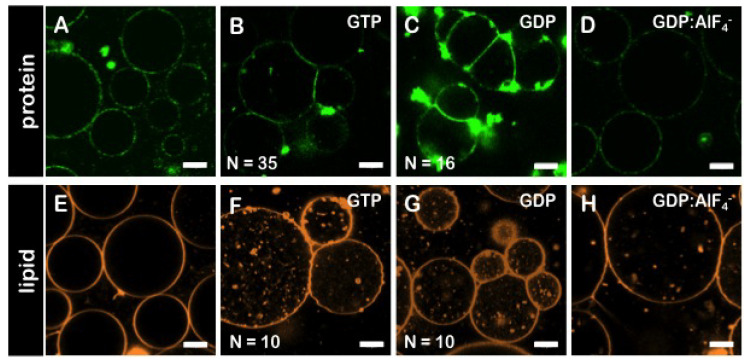
**Membrane adhesion of GUVs promoted by*****hs*****Mfn1**. Confocal fluorescence micrographs of GUVs incubated with solubilised *E. coli* inner membranes carrying *hs*Mfn1 and the α-Mfn1-488 antibody (top row, protein channel). (**A**) in the absence of nucleotide, (**B**) in the presence of GTP, (**C**) GDP and (**D**) GDP:AlF${}_{4}^{-}$. The GDP:AlF${}_{4}^{-}$ complex did not promote vesicle adhesion. Similar results were obtained with GUVs labeled with RhoPE in the absence of α-Mfn1-488 and incubated with solubilised Mfn1-IMVs (bottom row, lipid channel). (**E**) in the absence of nucleotide, (**F**) in the presence of GTP, (**G**) GDP and (**H**) GDP:AlF${}_{4}^{-}$. The lipid composition of GUVs was POPC/DOGS-NTA 80/20 mol% ratio. Scale bars are 10 μm. Each condition was repeated independently at least 3 times.

Upon the incubation of *hs*Mfn1-GUVs with GTP and MgCl_2_ at a final concentration of 10 mM and 2.5 mM respectively, we observed individual and multiple formation of adhesion areas when GUVs came into proximity (Figure 4A,B). The incubation of *hs*Mfn1-GUVs with GDP and MgCl_2_ also induced the formation of individual and multiple adhesion events of *hs*Mfn1-GUVs (Figure 4C). These findings suggested that under both conditions, we might look at the GDP-bound state of *hs*Mfn1. To confirm this hypothesis, we performed the same experiment in the presence of the GDP:AlF${}_{4}^{-}$ (Figure 4D). GDP:AlF${}_{4}^{-}$ bound to the nucleotide binding site of Mfn1 where the fluoride molecule mimicked the γ-phosphate of GTP and and the protein remains trapped in the GTP transition state [40]. As expected, we did not observe any significant adhesion of vesicles (Figure 4D). This result indicated that the hydrolysis GTP was important for membrane adhesion. Crystal stuctures showed that the Mfn1 dimer was only formed in the presence of GDP:AlF${}_{4}^{-}$ or GDP:BeF${}_{3}^{-}$[41]. GTP- or GDP-bound Mfn1 crystals resided as monomers and were indistinguishable from the *apo*-Mfn1 [41]. If present, the reported GDP:AlF${}_{4}^{-}$ Mfn1 dimer [17] must have been present in a *cis* interaction originating from the same membrane surface. This dimer was not proficient in membrane adhesion. The hydrolysis of GTP unlocked the transition state and induced a 77° rotation of the GTPase-domain re-orienting the Mfn1 binding interface [17].

To exclude that the vesicle adhesion was mediated by the presence of the Mfn1-antibody, we performed the assay in the absence of α-Mfn1-488 antibody, but labelled the GUV membrane with the fluorescent lipid probe Rh-PE. The presence of GTP or GTP also resulted in the adhesion of single and multiple events whereas the incubation with GDP:AlF${}_{4}^{-}$ did not promote adhesion between apposing vesicles (Figure 4E–H). However, the addition of GTP or GDP produced the formation of lipid fluorescent aggregates inside GUVs. Very recently, the ability of the HR1 of Mfn1 to induce liposome fusion in vitro has been shown. The fusion activity was associated to its capacity to destabilise membranes through its insertion into the lipid bilayer. This ability might explain the observed membrane destabilization here [42]. Finally, to rule-out that the observed membrane adhesion could be mediated by unspecific interactions between other proteins originating from the *E. coli* inner membranes, we incubated with 10 mM GTP both the solubilised Mfn1-IMVs incubated with POPC GUVs (in the absence of DOGS-NTA) (Figure 3 bottom row, *hs*Mfn1) and the solubilised native IMVs with POPC/DOGS-NTA GUVs (Figure 3 bottom row, *Ec*IMVs). No adhesion events were observed in those samples as *hs*Mfn1 was either absent or not able to bind to the GUV surface. The presence of Mfn1-IMVs between two adhered vesicles could trigger vesicle association through histidine-tag/Ni-NTA interactions from both IMV-GUV interfaces, as long as there are multiple Mfn1 in IMVs. However the histidine-tag/Ni-NTA interaction did not depend on GTP hydrolysis and we never observed membrane adhesion of GUVs incubated with Mfn1-IMVs in the absence of nucleotide (see Figure 4A,E). Thus we can conclude that the histidine-tag/Ni-NTA interaction from an hypothetical GUV–IMV–GUV arrangement can be ignored.

### 3.7. GTP-Dependent Change of the Average Diameters Mfn1-LUVs

To obtain an estimate of particle size of our preparations, we determined the average diameters of POPC/DOGS-NTA LUVs (80:20 mol%) after incubation with Mfn1-IMVs in the presence of GTP by means of dynamic light scattering (DLS) on a ZetaPlus Particle Analyzer (Brookhaven Instruments). Pure POPC/DOGS-NTA LUVs were found to have a mean diameter of 98 ± 1 nm at 20 °C. Upon incubation with Mfn1-IMVs, the mean *hs*Mfn1-LUV diameter increased to 111 ± 1 nm. The addition of GTP to a final concentration of 10 mM increased the mean *hs*Mfn1-LUV diameter within 1 h from 121 ± 2 nm to 145 ± 3 nm. This observed increase in size of ∼32% supports the Mfn1-mediated adhesion of LUVs upon GTP hydrolysis. In addition, the polidispersity index found for all samples (PDI < 0.2) indicated that the samples were monodisperse.

### 3.8. The Adhesion Strength Mediated by GDP-Mfn1 Is Comparable to GTP-Mfn1

We measured the nucleotide-dependent adhesion strength of *hs*Mfn1 proteins in vesicle pairs. The quantification of the adhesion strength was based on geometrical considerations from the shape of adhered vesicles regardless the nature of the binding molecules. Two adhesion regimes were described depending on the “tension state” of adhered vesicles [43]. In the weak adhesion regime ($E_{adh}<10^{-6}$ J/m^2^), the shape of the vesicles results from the interplay between adhesion and bending energy and the septum between vesicles exhibit a curved shape. In the strong adhesion regime ($E_{adh}>10^{-6}$ J/m^2^), the bending rigidity is negligible and the vesicles form a shape that is very close to a spherical cap. In our case, the curved contacts displayed by vesicles (see Figure 4) were consistent with a weak adhesion scenario. Depending on the adhesion strength, different approaches were used to obtain the binding strength of adhering molecules [33,44,45]. None of them provided information on the origin of the physical interactions between molecules but have been widely used to determine the adhesion energy promoted by very different systems based on polymer-induced osmotic depletion [44], ligand–receptor interaction [46] or electrically-induced adhesion [45].

**Figure 5:**
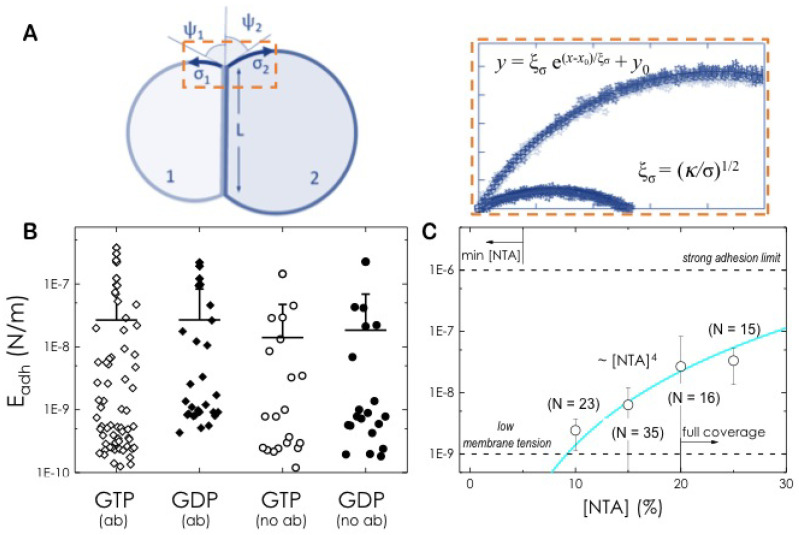
**Quantification of adhesion strength**. (**A**) Schematic diagram of two GUVs in adhesive contact. Contact angles are denoted by $\psi_{1},\psi_{2}$ and $\sigma_{1}$ and $\sigma_{2}$ are the lateral tensions. The vesicle contour was obtained from the pixels with maximum fluorescence intensity (stars in the zoomed area) and fitted to the function $y=\xi_{\sigma }^{\left(x-x_{0}\right)/\xi_{\sigma }}+y_{0}$ from the rim of the contact area up to the asymptote in the free membrane. Lateral tension values were directly calculated using the correlation length $\xi_{\sigma }={\left(\kappa /\sigma \right)}^{1/2}$, where $\kappa =10\kappa_{B}$T is the reported bending rigidity of POPC-containing GUVs [34]. (**B**) Adhesion strength of POPC/DOGS-NTA GUVs (80:20 mol%) carrying *hs*Mfn1 proteins under GTP (N = 35, 10) or GDP (N = 16, 10) incubation and the presence (squares) or absence (circles) of α-Mfn1-488 respectively. (**C**) Adhesion strength of POPC/DOGS-NTA GUVs binding *hs*Mfn1 proteins under GDP incubation and as a function of the concentration of NTA. In parenthesis, the number of adhesive events analysed. Data were fitted to Equation (6).

We analysed [33] multiple adhesive events and measured the contact angles $\psi_{1}$, $\psi_{2}$ between adhering GUVs (see Figure 5A for details). The bending modulus κ of the lipid bilayers, which is needed to estimate the lateral tensions (see Equation (1)), were taken from the reported values of POPC vesicles [34]. Finally, we calculated the adhesion energy using the experimental values of the pair averages of angles and tensions of adhering vesicles using Young’s Equation (1). We measured an adhesion strength of $E_{adh}=\left(2.7\pm 0.7\right)10^{-8}$ J/m^2^ upon GTP incubation that remained unchanged to $E_{adh}=\left(2.7\pm 0.5\right)10^{-8}$ J/m^2^ when incubated with GDP (Figure 5B). Although the presence of the α-Mfn1-488 alone was not able to artificially tether the GUVs and produce the adhesion interface (see Figure 3, top row and Figure 4A), it is well known that at high concentration and close vicinity immunoglobulins can induce “artificial” protein-protein interaction or contact [47]. To estimate the effect of the labelled antibody on the adhesion energy of GUVs we also quantified the adhesion strength in the presence of GTP or GDP but in the absence of Mfn1-antibody. We obtained $E_{adh}=\left(1.4\pm 0.3\right)10^{-8}$ J/m^2^ and $E_{adh}=\left(1.8\pm 0.5\right)10^{-8}$ J/m^2^ respectively (Figure 5B). Although slightly lower, the obtained values are of the same order to that obtained in the presence of Mfn1-antibody. These results exclude a significant contribution of the labelled antibody to the adhesion of vesicles.

### 3.9. Binding Cooperativity of Mfn1-Mediated Membrane Adhesion

In general, binding cooperativity during membrane adhesion resulted from a decrease in the roughness of the lipid bilayers with the increasing number of ligand-receptor bonds. These interactions constrained the membrane undulations and facilitate the binding of additional receptor and ligand molecules [22]. A direct approach to alter the adhesion strength was used to confirm and extend this scenario. We titrated the concentration of the surface-bound *hs*Mfn1 through the DOGS-NTA linker molar ratio and measured the adhesion strength $E_{adh}$ for GUVs containing 10, 15, 20 and 25 %mol DOGS-NTA in the presence of GDP. We did not observe vesicle adhesion below a critical concentration of 10% mol of DOGS-NTA. A minimal amount of NTA linkers is required to achieve stable binding of his-tagged molecules through the formation of multivalently bound species and prevent their desorption [36,48]. In general, vesicles carrying more DOGS-NTA lipids exhibit higher specific adhesion strength (Figure 5C). Increasing lateral tension through the cooperative binding mechanism increases the adhesion strength $E_{adh}$ up to approx. 10${}^{-7}$ J/m^2^. From the fit to $E_{adh}\propto {\left(K_{2D}\right)}^{2}{\left[NTA\right]}^{4}$ (Equation (6)) we obtained a $K_{D}^{{}^{\prime }}\approx 0.1$ nm^2^ per molecule. This value was three orders of magnitude lower than the two-dimensional binding equilibrium constant obtained for high affinity molecules such as antibodies [49,50]. Nevertheless, the attractive interactions promoted by Mfn1 molecules were strong enough to counterbalance the undulation forces from membrane fluctuations but not sufficient to drive complete membrane fusion.

### 3.10. Lipid Mixing Assay of GUVs Bearing Adhesion

**Figure 6:**
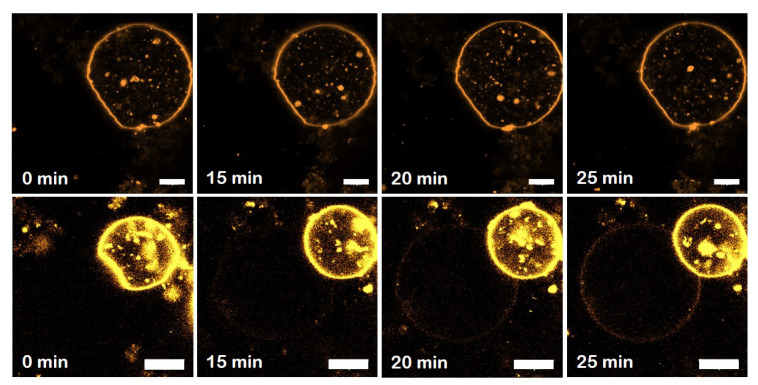
**Time evolution of lipid mixing assay in GUVS bearing Mfn1-induced adhesion.** Lipid mixing was monitored when fluorescence intensity was observed on the non-labelled vesicle after adhesion of two GUVs, one of them labelled with Rh-PE (0.5%mol). In 90% cases (N = 10) no fluorescence redistribution between vesicles was observed (**top row**). Only in one case, a fluorescent signal appeared in the non-labelled vesicle, suggesting lipid mixing (**bottom row**). Note that the equilibrium was only reached after 20–30 min. Scale bars are 10 μm.

Complete membrane fusion proceeds through a series of intermediate stages that include membrane approach and deformation; membrane adhesion; hemifusion, leading to lipid mixing of outer monolayers; and formation and growth of a fusion pore, leading to mixing of the two aqueous compartments [3]. To ascertain if the Mfn1-mediated adhesion of GUVs triggered the formation of hemifused vesicles we monitored the fluorescence density redistribution between two adhering vesicles. One of the vesicles contained 0.5 mol% Rh-PE as a fluorescent membrane label and the other vesicle had no fluorescence. After adhesion only a weak fluorescent rhodamine signal was monitored on non-fluorescent vesicles (Figure 6, top row). The fluorescence gain measured in non-fluorescent vesicles was 2–10 times lower than the theoretical ratio displayed for vesicles enduring hemifusion [51]. Nevertheless, we found only that 10% of cases showed lipid redistribution (Figure 6, bottom row). The absence of lipid exchange indicates the lack of lipid hemifusion structures, a process that is key for membrane fusion [51]. Protein-independent lipid mixing and subsequent membrane fusion may be only efficient for highly curved vesicles with a diameter of 100–200 nm [42] or the result of membrane destabilisation by the presence of non-bilayer lipids [52,53]. To test the role of lipid composition, we included up to 50 mol% of the fusogenic dioleyl- phosphatidylethanolamine (DOPE) [54] into the bilayer composition. We were not able to observe any fusion event or GUV collapse evolving from two adhering vesicles into one spherical vesicle (data not shown). Moreover, the steric hindrance provided by the existence of Mfn1-IMVs between adhering vesicles might have prevented the formation of the hemifusion state required for a complete membrane fusion. As we could not discard a lipid transfer from membranous structures present within the bulk solution, the promotion of membrane hemifusion by *hs*Mfn1 requires further investigation using micromanipulated vesicles [51].

## 4. Discussion

Mitochondria are highly dynamic and interconnected organelles that undergo continuous fusion and fission events to ensure a balanced mitochondrial homeostasis [55] and guarantee the constant supply of biochemical energy in form of ATP [56,57]. Mitochondrial dynamics are controlled by the dynamin-related protein 1 (Drp1), Mfn1 and Mfn2 and Opa1, all dynamin like proteins (DLPs) and members of the of the Dynamin protein superfamily that catalyse diverse membrane remodelling in different organelles [58]. Drp1 acts during the fission event, whereas Mfn1, Mfn2 and Opa1, localised in the OMM and the IMM respectively, operate upon the fusion event. During membrane fusion, DLPs are believed to share common mechanical features derived from dynamin protein including protein dimerization and a large conformational change within the monomers upon the binding and hydrolysis of GTP [15,59]. Nonetheless, the DLP family GTPases have been reported to have a rather low affinity for GTP nucleotides when compared with small regulatory GTPases of the Ras family, where the balanced action of associated guanine nucleotide-exchange factors (GEF) or guanine nucleotide-activating proteins (GAP) control their high affinity GTP binding and hydrolysis [60].

The available crystal structure of the Mfn1 deletion mutant that lacked both TMSs (an Mfn1${}_{IM}$) resulted in dimerisation in the presence of GDP:AlF${}_{4}^{-}$ but remains monomeric in the absence of nucleotide and in presence and of either GTP or GDP [41]. GDP:AlF${}_{4}^{-}$ is widely used in functional and crystallographic studies of GTP binding proteins [61], mimicking the GTP structure where the AlF${}_{4}^{-}$ positioned as the γ-phosphate of GTP [62] and assembleed into a ternary complex with an enhanced thermodynamic stability relative to the Mg-GDP-protein complex [63]. The displacement of the Mg^2+^ reduced the GTP hydrolysis and allowed it to trap the transient GTP induced conformational state of the GTP binding protein [64]. The binding and hydrolysis of GTP only took place upon dimer formation, where the GTP transition state during GTP hydrolysis was shown to be stabilised through the presence of additional sodium or potassium ions within the active site [65]. The dimerization of Mfn1 was mainly mediated via a hydrophobic cluster and salt bridges within the GTPase-domain interface across the nucleotide-binding, thus if GTP hydrolysis triggered, among other processes, mitochondrial fission and fusion, it should be controlled to not squander GTP by continuous hydrolysis [66].

**Figure 7:**
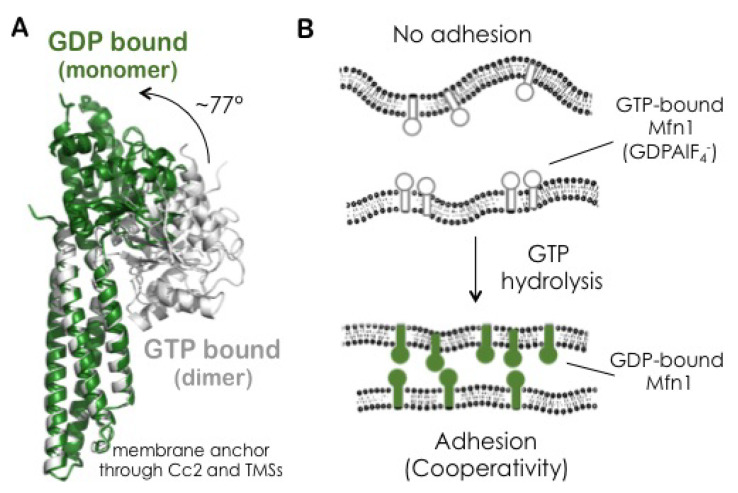
**Working model for the role of Mfn1 on the adhesion of lipid membranes.** (**A**) Crystal structure of MFN1 construct (without Cc2 and transmembrane domains; pdb 5GOE (green; [17,75]) and pdb 5YEW (grey; [17]). The conformational change of the GTPase domain relative to the first coiled-coil domain HR1 is indicated with an arrow. (**B**) A possible scenario for Mfn1-mediated membrane adhesion. According to the available literature, our data suggest that the Mfn1 proteins resides in a *cis*-dimer GTP-bound state that is disrupted upon GTP hydrolysis. GTP hydrolysis produces a conformational change from a compact “GTP-bound” *cis*-dimer to an elongated “GDP-bound” monomeric state [17]. These monomers are now able to interact in trans with other extended proteins GDP-bound Mfn1 proteins from adjacent membranes. Interaction affinity and the adhesion strength is primarily determined by the distance between the adjacent undulating membranes. The formation of *trans*-dimers limits the local membrane undulations and promotes the formation of additional trans “GDP”-Mfn1 interactions. In this way, the adhesion force depends in a cooperative manner depending on the local protein concentration and the strength of membrane undulation. At low protein coverage, the repulsive membrane undulations prevent membrane adhesion (Top), whereas a high protein coverage leads to a cooperative binding of the lipid bilayer counterparts (Bottom). With our setup, we do not observe any fusion event, therefore we suggest that the complete membrane fusion requires additional factors such as the presence of the Mfn2.

The presence of a control mechanism could be envisaged for the activity of the Mfn1 and Mfn2 proteins. They are very similar in sequence and predicted tertiary structure, but the purified proteins were shown to differ in their GTP hydrolysis rates during fusion. Mitochondria that only carried Mfn1 or Mfn2 displayed a relatively low fusion efficiency [11,67]. Mfn2 exhibited a greater binding affinity for GTP than Mfn1, but Mfn1 exhibited a higher GTP hydrolyzing activity [16]. This is an indication that membrane fusion needed the right stoichiometry between Mfn1 and Mfn2. This might be achieved through a pre-translational regulation. The *m*RNA levels of both Mfn1 and Mfn2 varied among different tissues. Mfn1 was preferentially expressed in heart, liver, pancreas, adrenal glands, and testis [68,69],whereas Mfn2 was more abundant in heart, skeletal muscle, brain, adrenal glands and brown adipose tissue [13,70,71]. Mutations in Mfn2 were associated with the neurodegenerative disorder Charcot–Marie–Tooth syndrome type 2A (CMT2A) and some of these mutants were unable to support fusion alone [72]. However the combination of these mutants in *cis* (on the same membrane) or in *trans* (on opposite membranes) with wild-type Mfn1 was able to rescue the mitochondrial phenotype and CMT2A [72,73]. Recently, a region within Mfn1 adjacent to the GTPase domain that extends into the HR1 domain called the mitofusin isoform-specific region (MISR) was identified [74]. Exchanging this domain between Mfn1 and Mfn2 highlighted its unique contribution to nucleotide-dependent self-assembly of Mfn1 and may have been involved in the regulation of the fusion activity of Mfn1 alone or in concert with Mfn2. In bacterial dynamin-like proteins (BDLPs), this region contains a hinge that mediates a switch from an open to a closed conformations of the protein [18]. In analogy to this switch, MISR was proposed to regulate the fusion events either directly as an interaction interface that promotes higher-order assembly of Mfn or indirectly through a nucleotide-dependent conformational changes, switching from a “rest” to an “active” state [74]. In particular, upon GTP hydrolysis, the Mfn1 monomers rotated ∼ 77° within their first HR1 domain giving rise to large conformational change that was interpreted as the power-stroke necessary for membrane fusion [17,75]. This large rotation disrupted the Mfn *cis*-dimers and produced extended GDP-bound monomeric Mfn1 proteins. These monomers did not permit the formation of Mfn1 *cis*-dimers due to a change in geometry and the displacement of their interaction interface [17] (Figure 7A).

The post-translational regulation of fusion activity through the modulation of a co-factor or the oligomeric state of the protein is a feature that seems to be conserved for other dynamin family members [76] as observed for atlastin (ALT), a membrane protein involved in the fusion of the endoplasmic reticulum (ER) [77,78].The presence of GTP induces a relatively weak ALT dimer that is strengthened by GDP:AlF${}_{4}^{-}$[79]. The precise timing and necessity of GTP hydrolysis might be triggered by the availability ions as K^+^ and Mg^2+^, shown to render the nucleotide-binding pocket within the GTP binding domain highly selective to promote nucleotide hydrolysis [41,75].

In relationship to the high complexity of the fusion event it is worth mentioning that next to the described mitochondrial fusion activity, Mfn2 was reported to be implicated in the direct interaction between mitochondria and the ER. So far, the claims on the ER-mitochondria tether are based on phenomenological observations giving rise to alternative interpretations that are heavily under debate [80]. The debate concentrates on the role of Mfn2 during the ER-mitochondria tether. On one side it is claimed that Mfn2 favours the ER-mitochondria tether [81,82] or rather prevents it where it functions as a negative regulator [83,84,85,86]. The meaning of these observations or their underlying molecular details are not yet understood, but another indication of the distinct roles of Mfn1 and Mfn2 during mitochondrial homeostasis.

Another important aspect that will be mentioned but not further discussed is the regulation of Mfn activity through the phosphrylation by the PINK1 phosphorylase that initiates mitochondrial recruitment for apoptosis. Phosphorylated Mfn2 that promotes mitochondrial mitophagy has no fusogenic activity [87]. A combination of phosphorylation, either promoting or preventing mitochondrial fusion, of Mfn and their resulting conformational changes determines the molecular behaviour of Mfn [88,89].

The fusion of individual mitochondria consists in the physical apposition of the organelles and the tethering of the adjacent OMMs that further progresses to their fusion which is then followed by the tethering and fusion of their IMMs [21]. The overall activation energy for spontaneous bilayer fusion, comprising all the intermediate stages, has been measured very recently [90]. The authors reported a value of 30 k*_B_*T, which is at the lowest range of previously estimated from theoretical models (20 to 150 k*_B_*T). From a thermodynamic point and as long as the membranes are close enough, a low energy input might trigger membrane fusion. In other words, membranes are ready to fuse if other forces, such as the steric repulsion from membrane fluctuations, would not prevent the approach of apposing membranes. The current picture of Mfn-promoted fusion is based on the direct conversion of the biochemical energy from GTP hydrolysis into mechanical movement; thus proteins are often seen as mechanical actuators that undergo large conformational upon nucleotide hydrolysis to exert unbalanced mechanical forces. The energy provided by the hydrolysis of one GTP molecule to GDP produces about 20 k*_B_*T. If we consider that only a 40–50% of this energy is transformed into mechanical force [91], the hydrolysis of only a few GTP molecules would provide sufficient energy to exceed the energy barrier required for membrane fusion. However, cryo-EM tomography studies show that the adhesion state previous to fusion in yeast is structurally maintained by tens of proteins spread over the contact region between apposing mitocondria, where the initial cluster of the Mfn-homolog Fzo1p [92] creates of a protein-denuded contact zone in both adhering membranes that allows the bilayers to contact and merge [92]. Considering a cross section for a single adhesion contacts in mitochondria of $10^{-2}-10^{-1}$μm^2^ [92]; the surface energy required for fusion would be 30k*_B_*T distributed over this area and $E_{adh}\approx 10^{-8}-10^{-7}$ J/m^2^. This range matches the experimental value obtained from our experiments. Moreover, also from the EM experiments, the distribution of 30 k*_B_*T over 10^2^ molecules leads to a binding interaction of ≈1 kJ/mol, which is the typical value for protein-protein surface interaction. In consequence, the energy input provided by a single protein is on the order of 1 k*_B_*T and only a cooperative mechanism involving multiple contacts would suppress the repulsive membrane undulations of lipid bilayer counterparts and thus overcome the activation energy barrier for fusion. The cooperative action of multiple *trans*-dimers was commented for Fzo1p, where the initial weak trans protein–protein interaction is strengthened to eventually lead to membrane fusion [92]. It is believed that Fzo1p is able to carry out both functions performed by Mfn1 and Mfn2 [12].

In our experiments (Figure 7B), no adhesion of adjacent Mfn1-GUVs is observed when incubated with GDP:AlF${}_{4}^{-}$ [93] (see Figure 4D,H). We propose Mfn1 to reside in a fusion incompetent state as the material behaves like bare GUVs. This state might correspond to the GTP transient state, structurally supported by a GTP-bound dimeric coformation of two parallel *cis*-arranged Mfn1 proteins within the same membrane[41]. Thermal shape fluctuations of the elastic membrane bilayer, with an amplitude of 100 nm, might act as a source of long-range interactions between *cis*-dimers as was observed for cadherin molecules [94]. Upon GTP hydrolysis, in the presence of GTP or GDP, the GTP-state turns into an adhesion competent GDP-state (Figure 4B,C,F,H). Now, Mfn1 dimers might undergo a large conformational change that leads to the extended GDP-bound monomeric state [17]. Upon the formation of *trans*-Mfn dimers, the amplitudes of the membrane fluctuations diminish and a stiffer membrane facilitates the formation of additional *trans*-Mfn dimers. We observe that for our set-up as the GUV adhesion and the adhesion strengths were correlated with the cooperative availability of Mfn1 *trans*-dimers within the adhesion interface Figure 5C [22].

A similar mechanism might occur in mitochondria and other organelles. According to the crystal structures of BDLP [18], mitochondrial apposition and tethering would solely be controlled by the dimerisation of the N-terminal GTPase domains. Though mutations within the functional GTPase domain interfered with mitochondrial fusion they did not affect mitochondrial tethering [72]. As a result mitochondrial aggregation inside the cell was observed [95]. Very recently it has been stressed that being a soluble protein [18], the GTPase dimerisation of the BDLPs do not represent the membrane bound conformation of the DLPs [18,19]. The available crystal structures of Mfn1 or Mfn1${}_{IM}$ [17] missing either their C-terminal domains or only the predicted TMSs respectively, have been arranged and modelled with respect to BDLP without considering the missing parts of the proteins. The C-terminal part as the TMSs have been assigned to be important for proper orientation and function of the protein. Early biochemistry indicated that the tethering and adhesion is initiated via an anti-parallel trans binding of the HR2 domain [95,96] and it was proposed that the C-terminus comprising this region faced to the cytosol [12]. However, recent biochemistry indicate that the C-termini of Mfn point to the mitochondrial intermembrane space [14] and thus challenges the topology model and the anti-parallel binding of the HR2 domain. Moreover, the importance of the TMS and the C-terminal HR2 domain for protein assembly and activity was demonstrated for the proposed mechanism of ER fusion catalyzed by the ATL protein [97,98]. Within the dynamin super family, the ATL protein is one of the closest relatives of Mfn. The ATL TMS forms an intramembrane hairpin that does not span the phospholipid bilayer [20] and Mfn-ATL chimeras carrying the ALT TMS were localised to the ER and promoted ER fusion [97,98]. Therefore, organelle specific information must be encoded in this region [74]. In addition, hydrophobic residues upstream to the Mfn TMS were shown to play a role in membrane targeting but not fusion [97,98]. Whether the conformational changes of the Mfn are the mechanical basis for mitochondrial tethering and fusion as their arrangement of Mfns into *cis* and *trans* dimers currently remains unclear [55].

## 5. Conclusions

We have studied the membrane adhering properties of Mfn1 specifically bound to the surface of giant lipid vesicles through the interaction between the histidine-tagged protein and NTA-lipids. The adhesion between protein-decorated vesicles was promoted upon incubation with GDP and GTP, whereas the GTP transition-state analogue GDP:AlF${}_{4}^{-}$ did not induce membrane remodelling. The adhesion strength mediated by Mfn1 is weak ($\approx 10^{-8}$ J/m^2^), but the binding of proteins from apposing membranes followed a cooperative behaviour as the NTA accessible sites were increased on the surface of lipid bilayers. Cooperative binding might act as a regulating mechanism to prevent an uncontrolled ready-to-fuse state of Mfn1. According to the current literature, we propose Mfn1 to change upon GTP hydrolysis, from a fusion incompetent dimeric state within the same membrane to an adhering GDP-bound monomeric state. As we did not observe fusion-like events, other factors might be required to orchestrate the Mfn-mediated fusion process in artificial models. Such claim forms a solid foundation for further research and requires the joint reconstitution of Mfn1 and other proteins such as Mfn2 in model lipid bilayers.

## References

[1] Martens S., McMahon H.T. (2008). Mechanisms of membrane fusion: Disparate players and common principles. Nat. Rev. Mol. Cell Biol..

[2] Chan D.C. (2006). Mitochondrial fusion and fission in mammals. Annu. Rev. Cell Dev. Biol..

[3] Chernomordik L.V., Kozlov M.M. (2008). Mechanics of membrane fusion. Nat. Struct. Mol. Biol..

[4] Ramachandran R., Schmid S. (2018). The dynamin superfamily. Curr. Biol..

[5] Antonny B., Burd C., De Camilli P., Chen E., Daumke O., Faelber K., Ford M., Frolov V.A., Frost A., Hinshaw J.E. (2016). Membrane fission by dynamin: What we know and what we need to know. EMBO J..

[6] Sousounis K., Haney C.E., Cao J., Sunchu B., Tsonis P.A. (2012). Conservation of the three-dimensional structure in non-homologous or unrelated proteins. Hum. Genom..

[7] Bramkamp M. (2012). Structure and function of bacterial dynamin-like proteins. Biol. Chem..

[8] McNew J.A., Sondermann H., Lee T., Stern M., Brandizzi F. (2013). GTP-dependent membrane fusion. Annu. Rev. Cell Dev. Biol..

[9] Legros F., Lombes A., Frachon P., Rojo M. (2002). Mitochondrial fusion in human cells is efficient, requires the inner membrane potential, and is mediated by mitofusins. Mol. Biol. Cell.

[10] Delettre C., Lenaers G., Griffoin J.M., Gigarel N., Lorenzo C., Belenguer P., Pelloquin L., Grosgeorge J., Turc-Carel C., Perret E. (2000). Nuclear gene OPA1, encoding a mitochondrial dynamin-related protein, is mutated in dominant optic atrophy. Nat. Genet..

[11] Chen H., Detmer S.A., Ewald A.J., Griffin E.E., Fraser S.E., Chan D.C. (2003). Mitofusins Mfn1 and Mfn2 coordinately regulate mitochondrial fusion and are essential for embryonic development. J. Cell Biol..

[12] Rojo M., Legros F., Chateau D., Lombes A. (2002). Membrane topology and mitochondrial targeting of mitofusins, ubiquitous mammalian homologs of the transmembrane GTPase Fzo. J. Cell Sci..

[13] Santel A., Fuller M.T. (2001). Control of mitochondrial morphology by a human mitofusin. J. Cell Sci..

[14] Mattie S., Riemer J., Wideman J.G., McBride H.M. (2018). A new mitofusin topology places the redox-regulated C terminus in the mitochondrial intermembrane space. J. Cell Biol..

[15] Daumke O., Roux A. (2017). Mitochondrial Homeostasis: How Do Dimers of Mitofusins Mediate Mitochondrial Fusion?. Curr. Biol..

[16] Ishihara N., Eura Y., Mihara K. (2004). Mitofusin 1 and 2 play distinct roles in mitochondrial fusion reactions via GTPase activity. J. Cell Sci..

[17] Yan L.M., Qi Y.B., Huang X.F., Yu C.T., Lan L., Guo X.Y., Rao Z.H., Hu J.J., Lou Z.Y. (2018). Structural basis for GTP hydrolysis and conformational change of MFN1 in mediating membrane fusion. Nat. Struct. Mol. Biol..

[18] Low H.H., Sachse C., Amos L.A., Lowe J. (2009). Structure of a bacterial dynamin-like protein lipid tube provides a mechanism for assembly and membrane curving. Cell.

[19] Low H.H., Löwe J. (2006). A bacterial dynamin-like protein. Nature.

[20] Betancourt-Solis M.A., Desai T., McNew J.A. (2018). The atlastin membrane anchor forms an intramembrane hairpin that does not span the phospholipid bilayer. J. Biol. Chem..

[21] Meeusen S., McCaffery J.M., Nunnari J. (2004). Mitochondrial fusion intermediates revealed in vitro. Science.

[22] Krobath H., Różycki B., Lipowsky R., Weikl T.R. (2009). Binding cooperativity of membrane adhesion receptors. Soft Matter.

[23] Sambrook J., Fritsch E.F., Maniatis T. (1989). Molecular Cloning. A Laboratory Manual.

[24] Baneyx F., Georgiou G. (1990). In vivo degradation of secreted fusion proteins by the Escherichia coli outer membrane protease OmpT. J. Bacteriol..

[25] Van der Does C., de Keyzer J., van der Laan M., Driessen A.J. (2003). Reconstitution of purified bacterial preprotein translocase in liposomes. Methods Enzym..

[26] Laemmli U.K. (1970). Cleavage of structural proteins during the assembly of the head of bacteriophage T4. Nature.

[27] Towbin H., Staehelin T., Gordon J. (1979). Electrophoretic transfer of proteins from polyacrylamide gels to nitrocellulose sheets: Procedure and some applications. Proc. Natl. Acad. Sci. USA.

[28] Lanzetta P.A., Alvarez L.J., Reinach P.S., Candia O.A. (1979). An improved assay for nanomole amounts of inorganic phosphate. Anal. Biochem..

[29] Bangham A.D. (1978). Properties and uses of lipid vesicles: An overview. Ann. N. Y. Acad. Sci..

[30] Lakowicz J.R. (1999). Principles of Fluorescence Spectroscopy.

[31] Almendro Vedia V.G., Natale P., Chen S., Monroy F., Rosilio V., López-Montero I. (2017). iGUVs: Preparing Giant Unilamellar Vesicles with a Smartphone and Lipids Easily Extracted from Chicken Eggs. J. Chem. Educ..

[32] Schneider C.A., Rasband W.S., Eliceiri K.W. (2012). NIH Image to ImageJ: 25 years of image analysis. Nat. Methods.

[33] Servuss R., Helfrich W. (1989). Mutual adhesion of lecithin membranes at ultralow tensions. J. Phys..

[34] Rodriguez-Garcia R., Arriaga L.R., Mell M., Moleiro L.H., Lopez-Montero I., Monroy F. (2009). Bimodal spectrum for the curvature fluctuations of bilayer vesicles: Pure bending plus hybrid curvature-dilation modes. Phys. Rev. Lett..

[35] Dembo M., Torney D.C., Saxman K., Hammer D. (1988). The reaction-limited kinetics of membrane-to-surface adhesion and detachment. Proc. R. Soc. Lond. B Biol. Sci..

[36] Nye J.A., Groves J.T. (2008). Kinetic Control of Histidine-Tagged Protein Surface Density on Supported Lipid Bilayers. Langmuir.

[37] Dumon-Seignovert L., Cariot G., Vuillard L. (2004). The toxicity of recombinant proteins in Escherichia coli: A comparison of overexpression in BL21(DE3), C41(DE3), and C43(DE3). Protein Expr. Purif..

[38] Knol J., Veenhoff L., Liang W.J., Henderson P.F., Leblanc G., Poolman B. (1996). Unidirectional reconstitution into detergent-destabilized liposomes of the purified lactose transport system of Streptococcus thermophilus. J. Biol. Chem..

[39] Mattjus P., Molotkovsky J.G., Smaby J.M., Brown R.E. (1999). A fluorescence resonance energy transfer approach for monitoring protein-mediated glycolipid transfer between vesicle membranes. Anal. Biochem..

[40] Scheffzek K., Ahmadian M.R., Kabsch W., Wiesmüller L., Lautwein A., Schmitz F., Wittinghofer A. (1997). The Ras-RasGAP Complex: Structural Basis for GTPase Activation and Its Loss in Oncogenic Ras Mutants. Science.

[41] Qi Y., Yan L., Yu C., Guo X., Zhou X., Hu X., Huang X., Rao Z., Lou Z., Hu J. (2016). Structures of human mitofusin 1 provide insight into mitochondrial tethering. J. Cell Biol..

[42] Daste F., Sauvanet C., Bavdek A., Baye J., Pierre F., Le Borgne R., David C., Rojo M., Fuchs P., Tareste D. (2018). The heptad repeat domain 1 of Mitofusin has membrane destabilization function in mitochondrial fusion. EMBO Rep..

[43] Seifert U., Lipowsky R. (1990). Adhesion of Vesicles. Phys. Rev. A.

[44] Sun Y., Lee C.C., Huang H.W. (2011). Adhesion and merging of lipid bilayers: A method for measuring the free energy of adhesion and hemifusion. Biophys. J..

[45] Steinkühler J., Agudo-Canalejo J., Lipowsky R., Dimova R. (2016). Modulating Vesicle Adhesion by Electric Fields. Biophys. J..

[46] Boulbitch A., Guttenberg Z., Sackmann E. (2001). Kinetics of membrane adhesion mediated by ligand-receptor interaction studied with a biomimetic system. Biophys. J..

[47] McPherson A., Gavira J.A. (2014). Introduction to protein crystallization. Acta Crystallogr. F Struct. Biol. Commun..

[48] Valiokas R., Klenkar G., Tinazli A., Reichel A., Tampé R., Piehler J., Liedberg B. (2008). Self-Assembled Monolayers Containing Terminal Mono-, Bis-, and Tris-nitrilotriacetic Acid Groups: Characterization and Application. Langmuir.

[49] Kuo S.C., Lauffenburger D.A. (1993). Relationship between receptor/ligand binding affinity and adhesion strength. Biophys. J..

[50] Steinkühler J., Różycki B., Alvey C., Lipowsky R., Weikl T.R., Dimova R., Discher D.E. (2018). Membrane fluctuations and acidosis regulate cooperative binding of ’marker of self’ protein CD47 with the macrophage checkpoint receptor SIRP*α*. J. Cell Sci..

[51] Heuvingh J., Pincet F., Cribier S. (2004). Hemifusion and fusion of giant vesicles induced by reduction of inter-membrane distance. Eur. Phys. J. E Soft Matter.

[52] Nishizawa M., Nishizawa K. (2010). Curvature-driven lipid sorting: Coarse-grained dynamics simulations of a membrane mimicking a hemifusion intermediate. J. Biophys. Chem..

[53] Rappolt M., Hickel A., Bringezu F., Lohner K. (2003). Mechanism of the Lamellar/Inverse Hexagonal Phase Transition Examined by High Resolution X-Ray Diffraction. Biophys. J..

[54] Lonez C., Lensink M.F., Kleiren E., Vanderwinden J.M., Ruysschaert J.M., Vandenbranden M. (2010). Fusogenic activity of cationic lipids and lipid shape distribution. Cell Mol. Life Sci..

[55] Dorn G.W. (2019). Evolving Concepts of Mitochondrial Dynamics. Annu. Rev. Physiol..

[56] Walker J.E. (2013). The ATP synthase: The understood, the uncertain and the unknown. Biochem. Soc. Trans..

[57] Scheffler I.E. (2008). Mitochondria.

[58] Ramachandran R. (2018). Mitochondrial dynamics: The dynamin superfamily and execution by collusion. Semin. Cell Dev. Biol..

[59] Gasper R., Meyer S., Gotthardt K., Sirajuddin M., Wittinghofer A. (2009). It takes two to tango: Regulation of G proteins by dimerization. Nat. Rev. Mol. Cell Biol..

[60] Hennig A., Markwart R., Esparza-Franco M.A., Ladds G., Rubio I. (2015). Ras activation revisited: Role of GEF and GAP systems. Biol. Chem..

[61] Jin Y., Molt R.W.J., Blackburn G.M. (2017). Metal Fluorides: Tools for Structural and Computational Analysis of Phosphoryl Transfer Enzymes. Top. Curr. Chem..

[62] Bigay J., Deterre P., Pfister C., Chabre M. (1985). Fluoroaluminates Activate Transducin-Gdp by Mimicking the Gamma-Phosphate of Gtp in Its Binding-Site. FEBS Lett..

[63] Macdonald T.L., Martin R.B. (1988). Aluminum ion in biological systems. Trends Biochem. Sci..

[64] Miller J.L., Hubbard C.M., Litman B.J., Macdonald T.L. (1989). Inhibition of transducin activation and guanosine triphosphatase activity by aluminum ion. J. Biol. Chem..

[65] Chappie J.S., Acharya S., Leonard M., Schmid S.L., Dyda F. (2010). G domain dimerization controls dynamin’s assembly-stimulated GTPase activity. Nature.

[66] Westermann B. (2010). Mitochondrial fusion and fission in cell life and death. Nat. Rev. Mol. Cell Biol..

[67] Chen H., Chomyn A., Chan D.C. (2005). Disruption of fusion results in mitochondrial heterogeneity and dysfunction. J. Biol. Chem..

[68] Eura Y., Ishihara N., Yokota S., Mihara K. (2003). Two mitofusin proteins, mammalian homologues of FZO, with distinct functions are both required for mitochondrial fusion. J. Biochem..

[69] Santel A., Frank S., Gaume B., Herrler M., Youle R.J., Fuller M.T. (2003). Mitofusin-1 protein is a generally expressed mediator of mitochondrial fusion in mammalian cells. J. Cell Sci..

[70] Zorzano A., Liesa M., Sebastian D., Segales J., Palacin M. (2010). Mitochondrial fusion proteins: Dual regulators of morphology and metabolism. Semin. Cell Dev. Biol..

[71] Bach D., Pich S., Soriano F.X., Vega N., Baumgartner B., Oriola J., Daugaard J.R., Lloberas J., Camps M., Zierath J.R. (2003). Mitofusin-2 determines mitochondrial network architecture and mitochondrial metabolism. A novel regulatory mechanism altered in obesity. J. Biol. Chem..

[72] Detmer S.A., Chan D.C. (2007). Complementation between mouse Mfn1 and Mfn2 protects mitochondrial fusion defects caused by CMT2A disease mutations. J. Cell Biol..

[73] Hoppins S., Edlich F., Cleland M.M., Banerjee S., McCaffery J.M., Youle R.J., Nunnari J. (2011). The soluble form of Bax regulates mitochondrial fusion via MFN2 homotypic complexes. Mol. Cell.

[74] Sloat S., Whitley B., Engelhart E., Hoppins S. (2019). Identification of a mitofusin specificity region that confers unique activities to Mfn1 and Mfn2. Mol. Biol. Cell.

[75] Cao Y.L., Meng S.X., Chen Y., Feng J.X., Gu D.D., Yu B., Li Y.J., Yang J.Y., Liao S., Chan D.C. (2017). MFN1 structures reveal nucleotide-triggered dimerization critical for mitochondrial fusion. Nature.

[76] Schrepfer E., Scorrano L. (2016). Mitofusins, from Mitochondria to Metabolism. Mol. Cell.

[77] Bian X., Klemm R.W., Liu T.Y., Zhang M., Sun S., Sui X., Liu X., Rapoport T.A., Hu J. (2011). Structures of the atlastin GTPase provide insight into homotypic fusion of endoplasmic reticulum membranes. Proc. Natl. Acad. Sci. USA.

[78] Byrnes L.J., Sondermann H. (2011). Structural basis for the nucleotide-dependent dimerization of the large G protein atlastin-1/SPG3A. Proc. Natl. Acad. Sci. USA.

[79] Hu J., Rapoport T.A. (2016). Fusion of the endoplasmic reticulum by membrane-bound GTPases. Semin. Cell Dev. Biol..

[80] Filadi R., Greotti E., Turacchio G., Luini A., Pozzan T., Pizzo P. (2017). On the role of Mitofusin 2 in endoplasmic reticulum-mitochondria tethering. Proc. Natl. Acad. Sci. USA.

[81] Naon D., Zaninello M., Giacomello M., Varanita T., Grespi F., Lakshminaranayan S., Serafini A., Semenzato M., Herkenne S., Hernández-Alvarez M.I. (2016). Critical reappraisal confirms that Mitofusin 2 is an endoplasmic reticulum–mitochondria tether. Proc. Natl. Acad. Sci. USA.

[82] De Brito O.M., Scorrano L. (2008). Mitofusin 2 tethers endoplasmic reticulum to mitochondria. Nature.

[83] Filadi R., Greotti E., Turacchio G., Luini A., Pozzan T., Pizzo P. (2015). Mitofusin 2 ablation increases endoplasmic reticulum–mitochondria coupling. Proc. Natl. Acad. Sci. USA.

[84] Cosson P., Marchetti A., Ravazzola M., Orci L. (2012). Mitofusin-2 independent juxtaposition of endoplasmic reticulum and mitochondria: An ultrastructural study. PLoS ONE.

[85] Leal N.S., Schreiner B., Pinho C.M., Filadi R., Wiehager B., Karlström H., Pizzo P., Ankarcrona M. (2016). Mitofusin-2 knockdown increases ER–mitochondria contact and decreases amyloid *β*-peptide production. J. Cell. Mol. Med..

[86] Wang P.T.C., Garcin P.O., Fu M., Masoudi M., St-Pierre P., Panté N., Nabi I.R. (2015). Distinct mechanisms controlling rough and smooth endoplasmic reticulum contacts with mitochondria. J. Cell Sci..

[87] Gong G., Song M., Csordas G., Kelly D.P., Matkovich S.J., Dorn G.W. (2015). Parkin-mediated mitophagy directs perinatal cardiac metabolic maturation in mice. Science.

[88] Rocha A.G., Franco A., Krezel A.M., Rumsey J.M., Alberti J.M., Knight W.C., Biris N., Zacharioudakis E., Janetka J.W., Baloh R.H. (2018). MFN2 agonists reverse mitochondrial defects in preclinical models of Charcot-Marie-Tooth disease type 2A. Science.

[89] Bhandari P., Song M., Chen Y., Burelle Y., Dorn G.W. (2014). Mitochondrial contagion induced by Parkin deficiency in Drosophila hearts and its containment by suppressing mitofusin. Circ. Res..

[90] Francois-Martin C., Rothman J.E., Pincet F. (2017). Low energy cost for optimal speed and control of membrane fusion. Proc. Natl. Acad. Sci. USA.

[91] Daumke O., Praefcke G.J. (2016). Invited review: Mechanisms of GTP hydrolysis and conformational transitions in the dynamin superfamily. Biopolymers.

[92] Brandt T., Cavellini L., Kühlbrandt W., Cohen M.M. (2016). A mitofusin-dependent docking ring complex triggers mitochondrial fusion in vitro. Elife.

[93] Wittinghofer A. (1997). Signaling mechanistics: Aluminum fluoride for molecule of the year. Curr. Biol..

[94] Fenz S.F., Bihr T., Schmidt D., Merkel R., Seifert U., Sengupta K., Smith A.S. (2017). Membrane fluctuations mediate lateral interaction between cadherin bonds. Nat. Phys..

[95] Koshiba T., Detmer S.A., Kaiser J.T., Chen H., McCaffery J.M., Chan D.C. (2004). Structural basis of mitochondrial tethering by mitofusin complexes. Science.

[96] Franco A., Kitsis R.N., Fleischer J.A., Gavathiotis E., Kornfeld O.S., Gong G., Biris N., Benz A., Qvit N., Donnelly S.K. (2016). Correcting mitochondrial fusion by manipulating mitofusin conformations. Nature.

[97] Huang X., Zhou X., Hu X., Joshi A.S., Guo X., Zhu Y., Chen Q., Prinz W.A., Hu J. (2017). Sequences flanking the transmembrane segments facilitate mitochondrial localization and membrane fusion by mitofusin. Proc. Natl. Acad. Sci. USA.

[98] Liu T.Y., Bian X., Sun S., Hu X., Klemm R.W., Prinz W.A., Rapoport T.A., Hu J. (2012). Lipid interaction of the C terminus and association of the transmembrane segments facilitate atlastin-mediated homotypic endoplasmic reticulum fusion. Proc. Natl. Acad. Sci. USA.

